# Attention or memory deficits? An HIV study in Shanghai using local and norm-based controls

**DOI:** 10.3389/fneur.2025.1555378

**Published:** 2025-05-09

**Authors:** Chuan Chen, Fengxiang Song, Chen Peng, Yuxin Shi, Dan-Chao Cai

**Affiliations:** Shanghai Public Health Clinical Center, Fudan University, Shanghai, China

**Keywords:** HIV, cognitive impairment, neurocognitive tests, norms, Shanghai

## Abstract

**Objective:**

HIV-associated neurocognitive disorder is a major complication in people living with HIV (PLWH), with standardized neuropsychological tests being essential for clinical diagnosis. However, the selection of healthy controls significantly influences the interpretation of test results. This study compares neuropsychological outcomes using two control methods, local HIV-negative controls versus decades-old norms, to evaluate neurocognitive impairments in Shanghai and assess the applicability of existing norms to contemporary populations.

**Methods:**

A total of 244 PLWH who attended the Shanghai Public Health Clinical Center between 2019 and 2024, along with 132 HIV-negative controls, participated in the study. Standardized neuropsychological tests covering seven cognitive domains were administered to provide a comprehensive cognitive function assessment. Neurocognitive impairments were defined by comparing them with either local controls or previously established norms.

**Results:**

After adjusting for age, sex, and education level, PLWH demonstrated significantly lower standardized scores compared to people living without HIV (PLWoH) in attention/working memory (49.2 ± 7.62 vs. 53.1 ± 6.34, Bonferroni-corrected *p* < 0.001). Executive function scores showed a marginal difference (49.3 ± 6.61 vs. 51.5 ± 6.22, Bonferroni corrected *p* = 0.052). No significant group differences were observed in other cognitive domains. Norm-based analyses identified impairments in attention/working memory and executive function among PLWH, with 12% and 13% impairment rates, respectively. In contrast, impairment rates in memory (32%), learning (20%), and motor (14%) were higher, although they did not differ significantly between PLWH and PLWoH.

**Conclusion:**

Neurocognitive impairments in PLWH from Shanghai primarily involve attention/working memory and executive function. However, norm-based analysis emphasized impairments in memory and learning, underscoring significant discrepancies between local controls and outdated norms. These findings underscore the limitations of relying on outdated norms for evaluating neurocognitive impairment and emphasize the importance of developing updated, localized norms for accurate diagnosis and effective interventions.

## 1 Introduction

People living with HIV (PLWH) are at a higher risk of experiencing cognitive impairment compared to HIV-seronegative controls (1, 2). However, the specific cognitive domains affected by HIV-associated brain injury vary significantly across studies. These discrepancies may arise from multiple factors, including differences in HIV-related clinical features and antiretroviral therapy regimens (1, 3, 4), as well as demographic factors such as age (5, 6). Region-related factors, such as language, culture, and socioeconomic environment, may also contribute (7, 8). Such factors have been shown to impact cognitive outcomes significantly in cross-cultural studies. For instance, a comparison study between China and the United States demonstrated that linguistic and cultural factors significantly influence neuropsychological test performance alongside the direct effects of HIV (9). These findings suggest that HIV may affect different cognitive domains in different PLWH populations, emphasizing the importance of analyzing cognitive impairment profiles within specific populations to enable effective cognitive screening and tailored treatment strategies.

When investigating cognitive impairment profiles across different PLWH populations, selecting an appropriate control group is crucial to ensure valid comparisons. Control groups must be carefully designed to address both demographic and region-related differences that can significantly influence cognitive outcomes (8). In practice, researchers typically rely on two types of controls: locally recruited HIV-negative control groups matched for demographic characteristics, with sample sizes comparable to the PLWH group, and alternatively, large-sample norms that align with the linguistic and cultural background of the PLWH population and encompass a broader range of demographics. While large-sample norms are often preferred due to their advantages in statistics and clinical settings (10, 11), they may fail to account for regional differences or rely on outdated data (12, 13). Outdated norms that have not been updated in over a decade may no longer reflect shifts in population characteristics, leading to inaccurate diagnoses or misinterpretation of cognitive profiles (14, 15). However, due to the significant effort required to establish or update norms, researchers often rely on unmatched or outdated norms, potentially compromising the accuracy of their findings.

In China, the use of population-specific norms for neuropsychological tests has increased in recent years, yet their applicability to current research contexts remains underexplored. Based on a review of 17 studies conducted between 2007 and 2024 on Mandarin-speaking HIV-infected populations, we observed a shift from using locally recruited HIV-negative control groups to relying on previously published norms. Among these, 11 studies published after 2016 employed norms described as “population-specific” (16). However, the origins of these norms were often unclear, with only one study specifying the city of origin (17). These norms, often derived from data collected more than a decade ago in rural (e.g., Anhui) and urban (e.g., Beijing) regions (18, 19), may no longer represent the current population. This reliance on potentially outdated norms raises concerns about their validity and the accuracy of findings in recent studies. For instance, Zhao and colleagues reported above-average performance in executive function among PLWH, raising questions about the appropriateness of the normative data used for comparison (20).

To evaluate whether previous norms are outdated for current populations, this study compares the results of neuropsychological tests using two control methods: locally recruited HIV-negative controls and norm-based standardized scores. We focused on the neurocognitive functions of HIV-infected individuals in Shanghai. By comparing PLWH receiving antiretroviral therapy with locally recruited controls, this study aims to identify the region-specific characteristics of neurocognitive impairments. Additionally, it assesses the applicability of large-sample norms from various urban areas, providing insights into whether localized norms need to be established or updated.

## 2 Materials and methods

### 2.1 Participants

We recruited 244 PLWH who attended the Shanghai Public Health Clinical Center between January 2020 and June 2024 and 132 HIV-seronegative individuals from the neighborhood as people living without HIV (PLWoH). All participants were aged 18 to 60. Exclusion criteria included: (1) medical or neuropsychiatric conditions that could confound the study (e.g., stroke, epilepsy, multiple sclerosis, cerebral palsy, Parkinson’s disease, dementia, schizophrenia, or bipolar disorder); (2) history of head injury or loss of consciousness lasting more than 30 min; (3) substance abuse defined as daily alcohol consumption exceeding 450 mL or drug use more than once a week in the past 30 days; (4) pregnant or postpartum individuals. The Ethics Committee of the Shanghai Public Health Clinical Center approved the study. Informed consent was obtained from all participants.

### 2.2 Neurocognitive tests

Systematic neuropsychological tests followed the Frascati criteria (21) and have been reported in our previous studies (17, 22), including 15 tests assessing seven cognitive domains. All the neuropsychological tests were carried out by experienced staff trained by experts from the HIV Neurobehavioral Research Center at the University of California San Diego. The center has published high-impact research on HIV-associated neurocognitive impairment (2, 23) and features worldwide international collaboration, including China (18). Additionally, the Beck Depression Inventory was used to assess depression symptoms, while the Patients Assessment of Own Functioning Inventory and the Instrumental Activities of Daily Living (modified version of the Lawton and Brody scale) were used to assess daily functions.

### 2.3 Normalization

Norm-based results were derived from a standardized dataset of 708 urban-dwelling participants in China (Beijing, *n* = 80; Hong Kong, *n* = 153; Shanghai, *n* = 72; and Kunming, *n* = 403) provided by the HIV Neurobehavioral Research Center. Part of this dataset was published previously (16). Raw test scores were converted to scale scores (mean = 10, SD = 3), then transformed into T-scores (mean = 50, SD = 10), accounting for age, sex, and years of education according to the norm. Domain T-scores were averaged across corresponding tests. Test and domain T-scores were used in the primary analyses to detect group differences with greater sensitivity.

As in previous studies, T-scores were further transformed into deficit scores ranging from 0 (normal) to 5 (severe impairment) to assess cognitive impairment. Deficit scores provide a clinically intuitive measure and are more commonly used in diagnostic settings. Deficit scores for seven cognitive domains were averaged across corresponding tests, and a global deficit score (GDS) was calculated by averaging the domain deficit scores. A GDS ≥ 0.5 was used to identify overall cognitive impairment, consistent with prior research (18). In addition, Frascati-defined classifications of neurocognitive impairment were determined based on norm-based deficit scores, depressive symptoms, and functional status, following established criteria (21).

Similar to norm-based scoring, we conducted local-sample-based scoring. The effects of age, sex, and years of education on the raw test scores were considered using general linear regression. A regression model was constructed using all samples from the current study rather than the large-sample norm data. Outliers for each measure, defined as values exceeding six standard deviations from the mean, were identified and removed iteratively before the regression analysis. The residuals from the regression models were subsequently standardized into T-scores. The T-scores were reversed for necessary measures to ensure that higher values correspond to better cognitive function or test performance. To evaluate the clinical implications of using different reference standards, Frascati classifications were also applied using deficit scores derived from sample-based T-scores.

### 2.4 Statistical analysis

There are several ways to define impairments in tests or cognitive domains in PLWH. In the norm-based approach, a domain deficit score equal to or greater than 1 was considered impaired for domain measures. Another way is to compare the norm-based T-scores with 50, which is the mean value of the T-scores in the normative sample, via one-sample *t*-tests. A test or domain was considered defective if the norm-based T-score was significantly lower than 50.

In the sample-based approach, sample-based T-scores were compared against the PLWoH using two-sample *t*-tests. A test or domain was considered defective if the sample-based T-score was significantly lower than PLWoH. For completeness, we also compared norm-based T-scores between the groups, although this approach may be less appropriate due to potential mismatches between historical norms and the current population. In addition, chi-squared tests were used to compare the proportions of individuals classified as impaired (i.e., domain deficit score ≥ 1) in each cognitive domain and for global deficit (GDS ≥ 0.5), based on norm-derived impairment definitions.

To assess whether historical norms were suitable for the current population, we also tested whether norm-based T-scores in PLWoH significantly differed from 50. This analysis served as a quality check for the applicability of the normative baseline.

## 3 Results

### 3.1 Demographic and clinical characteristics

A total of 244 PLWH and 132 PLWoH completed the neuropsychological tests. PLWH was significantly older than PLWoH (36.97 vs. 33.98 y, *t*[374] = 2.60, *p* = 0.010). There were significantly more male participants in the PLWH than in the PLWoH group (95% vs. 83%, *χ*^2^[1, 376] = 15.87, *p* < 0.001). The rate of self-reported depression did not differ in the two groups (15% vs. 17%, *χ*^2^[1, 376] = 0.04, *p* = 0.835). Three participants in the PLWH group reported a mild functional decline in daily activities. Owing to ethical and procedural limitations, we did not retrieve medical records directly but instead relied on participant-provided documentation. Among PLWH, 35% provided complete medical records, which are summarized in Table 1. There were no significant differences in sex distribution, age, or years of education between those with and without medical records.

**Table 1:** Demographic and clinical characteristics of the study sample.

Characteristic	PLWH		PLWoH	*p*-value*
*N*	244		132	
Male	232		109	
Male (%)	95%		83%	**0.000**
Age	36.97 ± 10.98		33.98 ± 9.98	**0.010**
Education	14.29 ± 2.62		14.38 ± 2.77	0.714
Minimal or no depression (%)	85%		83%	0.835
Medical records	Yes	No		
*N*	86	158		
Male	82	150		
Male (%)	95%	95%		1.000
Age	37.06 ± 9.97	36.94 ± 11.48		0.934
Education	14.26 ± 2.63	14.32 ± 2.61		0.863
Years since seroconversion	7.43 ± 5.08			
Nadir CD4, cells/μL	315 ± 225			
Nadir CD8, cells/μL	653 ± 332			
Max viral load, log_10_ copies/mL	2.99 ± 1.66			
Current CD4, cells/μL	562 ± 255			
Current CD8, cells/μL	872 ± 393			
Current viral load < 50 copies/mL (%)	97%			
Currently on antiretroviral therapy (%)	97%			

PLWH, people living with HIV; PLWoH, people living without HIV. *, bold indicates significant uncorrected *p*-values.

### 3.2 PLWH verse norms: norm-based deficits

The domain deficit scores and GDS were listed in Table 2. The most impaired cognitive domain in PLWH was memory, with 34% of PLWH having a domain deficit score equal to or greater than 1. The impairment rates of learning, executive function, motor, verbal, speed of information, and attention/working memory were 27, 20, 18, 15, 15, and 14%, respectively. As for global deficits, 26% of PLWH had a GDS equal to or greater than 0.5, while 46% of them met the diagnosis of asymptomatic neurocognitive impairment (ANI) according to the Frascati criteria. None of them met the diagnosis of mild neurocognitive disorder or HIV-associated Dementia.

**Table 2:** Norm-based deficit scores and impairment rates in PLWH and PLWoH.

Cognitive domain	PLWH		PLWoH		Statistics	
	Mean±SD	Imp.%	Mean±SD	Imp.%	*χ*^2^	*p^#^*
Learn	0.53 ± 0.78	27%	0.52 ± 0.83	23%	0.702	0.402
Memory	0.61 ± 0.86	34%	0.51 ± 0.69	29%	0.647	0.421
Motor	0.42 ± 0.87	18%	0.36 ± 0.84	14%	0.813	0.367
Executive	0.33 ± 0.53	20%	0.22 ± 0.38	11%	5.515	**0.019**
Verbal	0.20 ± 0.44	15%	0.16 ± 0.35	12%	0.655	0.418
SIP	0.28 ± 0.50	15%	0.17 ± 0.36	9%	2.788	0.095
Attn/WM	0.26 ± 0.55	14%	0.08 ± 0.27	5%	7.429	**0.006**
GDS	0.35 ± 0.39	26%	0.26 ± 0.30	18%	2.810	0.094
ANI		46%		36%	3.436	0.064

PLWH, people living with HIV; PLWoH, people living without HIV; SIP, speed of information processing; Attn/WM, attention/working memory; GDS, global deficit score; ANI, asymptomatic neurocognitive impairment; SD, standard deviation; Imp. %, percentage of impaired participants (domain deficit score ≥ 1, GDS ≥ 0.5). #, uncorrected *p*-values of chi-squared tests with bold indicating significant uncorrected *p*-values.

The norm-based T-scores in cognitive domains and tests were listed in Table 3. Norm-based T-score*s* were significantly lower than 50 in PLWH in five cognitive domains: learning (domain and all tests), memory (domain and all tests), motor (domain and the nondominant-hand Grooved Pegboard Test), executive function (Wisconsin Card Sorting Test), and attention/working memory (WMS-III Spatial Span). Conversely, norm-based T-scores were significantly higher than 50 in PLWH in verbal fluency (domain and Verbal Fluency Test on animals). As for the speed of information, PLWH was significantly lower than 50 in the WAIS-III Symbol Search and Color Trail Test 1 while significantly higher than 50 in the WAIS-III Digit Symbol, leading to an insignificant domain T-score. Statistical results are provided in Table 3.

**Table 3:** Comparison of cognitive domain and neuropsychological test scores: PLWH, PLWoH, and norms.

Cognitive domain^#^ Neuropsychological test	*T*-score				*p* value^*^		
	Norm-based		Sample-based		PLWH	PLWH	PLWoH
					versus	versus	versus
	PLWH	PLWoH	PLWH	PLWoH	Norm^†^	PLWoH^‡^	Norm^†^
***Learning***	***44.8***	***44.4***	***50.1***	***49.8***	***0.000***		***0.000***
HVLT - Learning	44.0	42.9	50.3	49.5	0.000		0.000
BVMT - Learning	45.7	46.0	50.0	50.0	0.000		0.000
***Memory***	***45.3***	***45.8***	***49.9***	***50.2***	***0.000***		***0.000***
HVLT - Delayed Recall	43.3	43.4	50.0	50.0	0.000		0.000
BVMT - Delayed Recall	47.2	48.1	49.7	50.5	0.001		
***Motor***	***47.6***	***48.3***	***49.9***	***50.2***	***0.005***		
Grooved Pegboard - NH	46.8	47.3	49.9	50.2	0.000		
Grooved Pegboard - DH	48.5	49.3	49.9	50.2			
***Executive function***	***49.0***	***50.9***	***49.3***	***51.5***		***0.052***	
WCST-64 Card Version	47.4	47.1	50.0	50.0	0.001		0.004
Stroop Test - Inconsistent	49.0	49.6	49.4	51.0			
Halstead Category Test	50.1	53.3	49.0	51.8			0.067
Color Trails 2	49.2	53.3	48.4	52.9		0.001	0.002
***Verbal fluency***	***51.8***	***50.9***	***50.3***	***49.4***	***0.020***		
Verbal fluency - Verb	51.2	49.3	50.5	49.1			
Stroop Test - Word	51.2	51.5	49.9	50.1			
Verbal fluency - Animals	52.9	51.9	50.5	49.1	0.000		
***SIP***	***50.3***	***52.2***	***49.4***	***51.1***			***0.007***
WAIS-III Symbol Search	47.0	49.3	49.0	51.8	0.000		
Stroop Test - Color	49.7	49.6	49.8	50.4			
Color Trails 1	47.9	50.3	49.3	51.3	0.022		
WAIS-III Digit Symbol	56.7	59.4	49.6	50.8	0.000		0.000
***Attn/WM***	***49.2***	***53.0***	***48.3***	***53.1***		***0.000***	***0.000***
WMS-III Spatial Span	47.7	51.9	48.5	52.7	0.011	0.003	
PASAT-50	50.6	54.1	48.1	53.6		0.000	0.000

PLWH, people living with HIV; PLWoH, people living without HIV; HVLT, Hopkins verbal learning test; BVMT, brief visuospatial memory test; NH, nondominant hand; DH, dominant hand; WCST, Wisconsin card sorting test; SIP, speed of information processing; Attn/WM, attention/working memory; PASAT, paced auditory serial addition test. #, domain results are presented in bold and italics. *, Bonferroni-corrected *p*-values, calculated by multiplying the uncorrected *p*-values by 26 to account for multiple comparisons presented in the table. †, Norm-based T-scores were compared against 50, the mean of the normative sample. ‡, Group comparisons between PLWH and PLWoH were conducted using sample-based T-scores. Comparable significance levels were observed using norm-based T-scores, with equal or slightly higher *p*-values.

### 3.3 PLWH versus PLWoH: sample-based deficits

**Figure 1:**
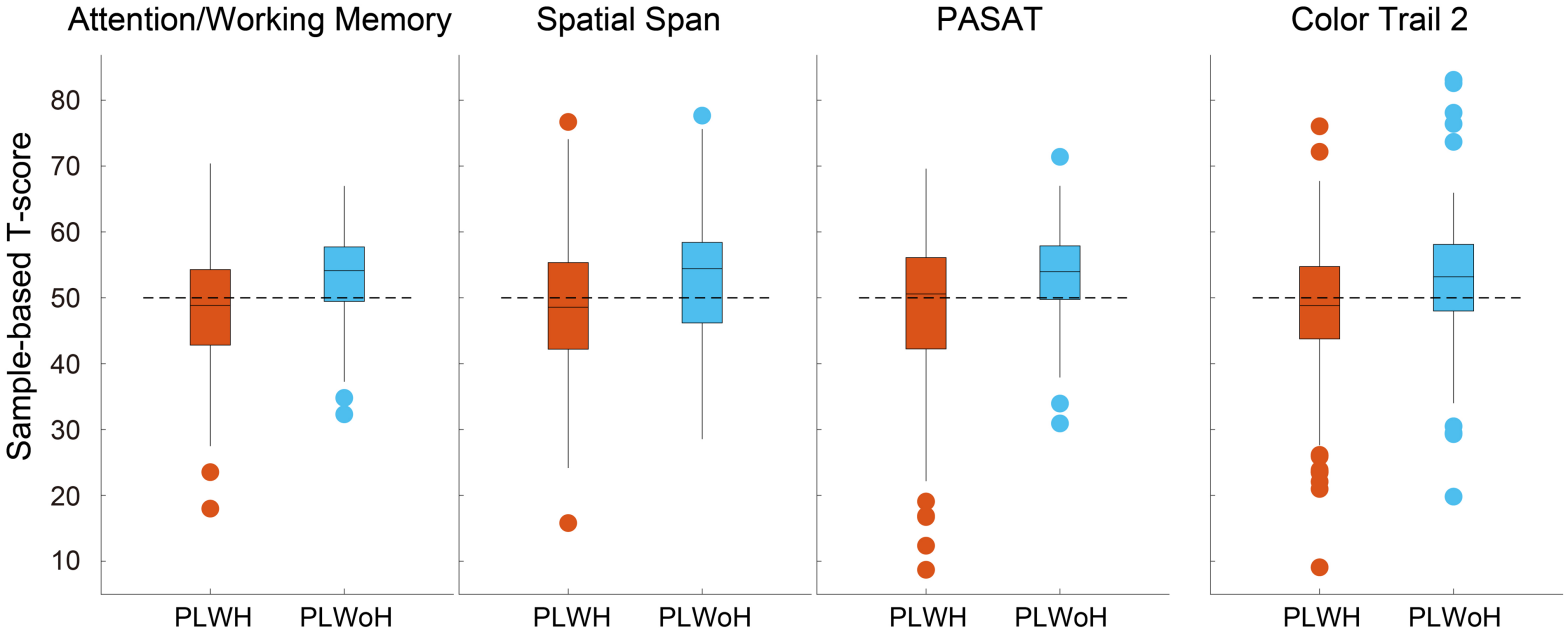
Cognitive domains and neuropsychological tests with significant group differences. Boxplots depict the sample-based T-scores for attention/working memory in PLWH (people living with HIV; red) and PLWoH (people living without HIV; blue), assessed through Spatial Span and Paced Auditory Serial Addition Test (PASAT). A significant group difference was also observed in Color Trail 2, which evaluates executive function. The dashed horizontal line represents the mean sample-based T-score, which is 50. Outliers are shown as individual points.

The central analysis of the sample-based group comparison was based on sample-based T-scores, which were calculated based on the whole sample of the current study. PLWH was significantly lower than PLWoH in the T-scores of attention/working memory [(48.3 ± 8.28) vs. (53.1 ± 6.25), Bonferroni-corrected *p* < 0.001]. Significant group differences were revealed in both tests used to assess attention/working memory (WMS-III Spatial Span, 48.5 ± 9.96 vs. 52.7 ± 9.55, Bonferroni-corrected *p* = 0.003; Paced Auditory Serial Addition Test, 48.1 ± 10.90 vs. 53.6 ± 6.78, Bonferroni-corrected *p* < 0.001). The group differences in Color Trail Test 2 were also significant [(48.4 ± 9.97) vs. (52.9 ± 9.44), corrected *p* = 0.001], resulting in marginally significant group differences in the domain of executive function [(49.3 ± 6.61) vs. (51.5 ± 6.22), corrected *p* = 0.052]. The distribution of sample-based T-scores with significant group differences are displayed in Figure 1. No significant group differences were revealed in the other five cognitive domains. The average sample-based T-scores of all domains and tests are provided in Table 3.

A secondary analysis using norm-based T-scores yielded a similar pattern of results. Group differences in attention/working memory remained significant (49.2 ± 7.58 vs. 53.1 ± 6.31, corrected *p* < 0.001), as did those in the component tests: WMS-III Spatial Span (47.7 ± 9.96 vs. 52.0 ± 9.50, corrected *p* = 0.002) and the Paced Auditory Serial Addition Test (50.6 ± 9.32 vs. 54.1 ± 6.73, corrected *p* = 0.005). Group differences also persisted in Color Trail Test 2 (49.2 ± 10.04 vs. 53.5 ± 9.97, corrected *p* = 0.002). Consistent with these findings, comparison of domain deficit scores, which are calculated from norm-based T-scores, also showed more PLWH were impaired than PLWoH in attention/working memory (14% vs. 5%, *χ*^2^[1, 320] = 7.429, uncorrected *p* = 0.006) and executive function (20% vs. 11%, *χ*^2^[1, 226] = 5.515, uncorrected *p* = 0.019).

### 3.4 PLWoH verse norms

**Figure 2:**
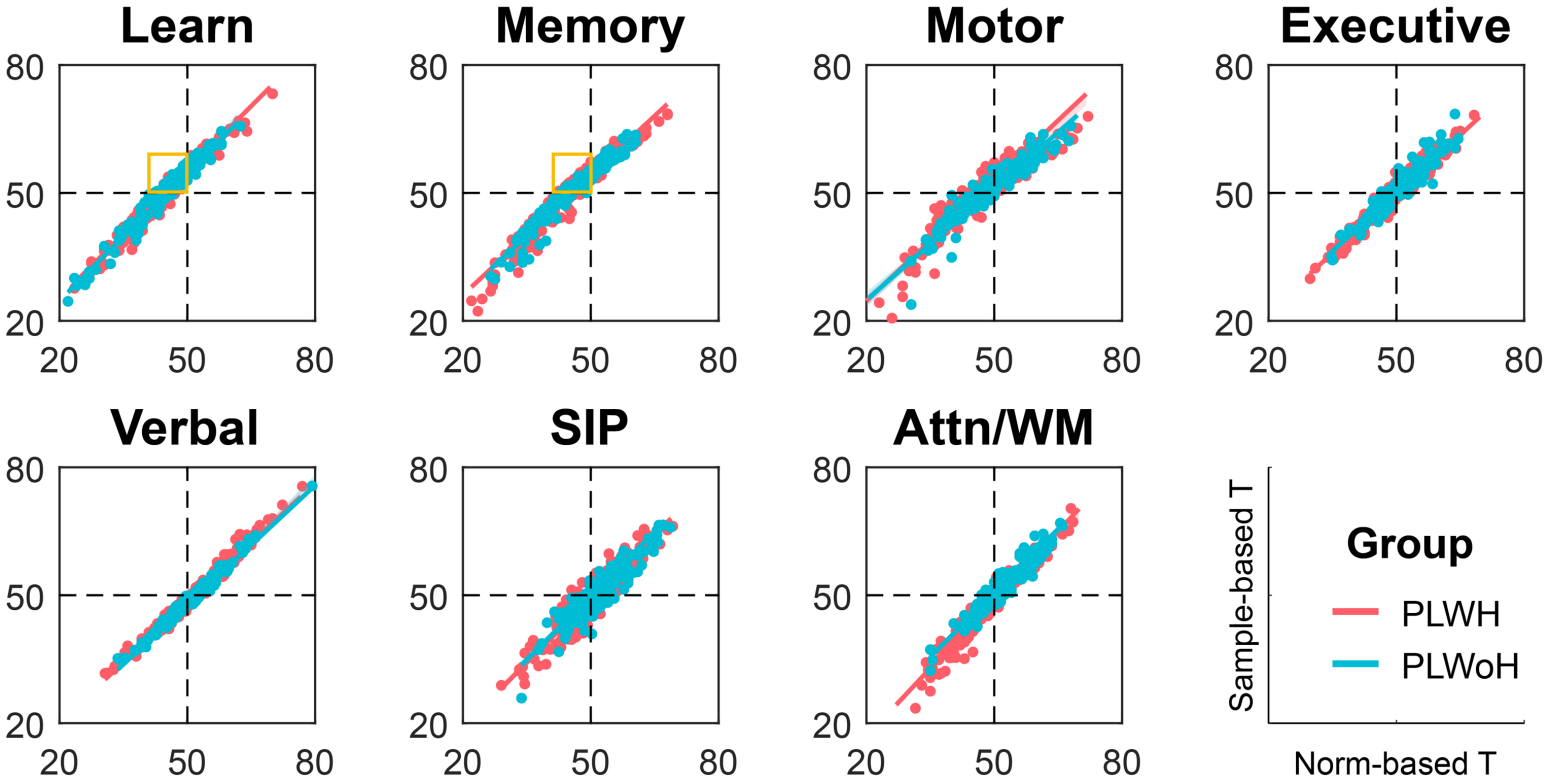
Norm-based and sample-based T-scores in seven cognitive domains. Orange boxes highlight individuals with under-average cognition according to norm-based T-score (< 50 on the horizontal axis) and above-average cognition according to sample-based T-score (> 50 on the vertical axis). SIP, speed of information processing; Attn/WM, attention/working memory; PLWH, people living with HIV; PLWoH, people living without HIV.

For global deficits, 18% of PLWoH had a GDS greater than 0.5, while 36% of them have ANI. As for domain deficits, the PLWoH sample in the current study performed worse than the norms (T-score*s* < 50) in memory (domain and Hopkins Verbal Learning Test) and learning (domain and all associated tests), with a domain impairment rate of 29 and 23%, respectively. In contrast, the same group outperformed the norms in SIP (domain and WAIS-III Digit Symbol) and attention/working memory (domain and Paced Auditory Serial Addition Test), with a domain impairment rate of 9 and 5%, respectively. As for the executive function, PLWoH was significantly lower than 50 in the Wisconsin Card Sorting Test while significantly higher than 50 in the Color Trails Test 2, leading to an insignificant domain T-score (domain impairment rate = 11%). The current sample of PLWoH exhibited similar performances as the norms in motor (domain impairment rate = 14%) and verbal fluency (domain impairment rate = 12%). Statistical results are provided in Table 3. These systematical differences between PLWoH and norms were reflected in the scatter plots where norm-based T-scores were plotted against sample-based T-scores (Figure 2). Among participants classified as impaired or under-average according to the norms, 57% (17 out of 30) in the learning domain and 66% (25 out of 38) in the memory domain were considered normal based on local controls in the current study. This contrast was also reflected in the Frascati-defined classifications, with ANI rates decreasing from 46 to 6% in PLWH and from 36 to 1% in PLWoH when applying sample-based rather than norm-based scoring.

## 4 Discussion

Compared to PLWoH from the same region, PLWH in Shanghai primarily exhibited neurocognitive impairments in attention/working memory and executive function, as measured by the Paced Auditory Serial Addition Test, Spatial Span, and Color Trail Test 2. However, when compared to norms derived from several Chinese cities decades ago, impairment rates in PLWH were highest in memory, learning, and motor functions. These findings highlight the impact of reference group selection on interpreting neurocognitive impairment profiles and underscore the need for appropriate baselines.

The discrepancies between conclusions drawn using these two references stem from differences between the existing norms and the local HIV-negative controls in this study. Specifically, the PLWoH group in this study outperformed historical norms in attention/working memory and executive function. While the current PLWH group performed similarly to the norms, their performance was reduced relative to the demographically matched local controls. This pattern explains why group differences were significant when comparing PLWoH to the norms, but not when comparing PLWH to the same norms, especially in tasks such as the PASAT. In contrast, PLWoH in the current study underperformed the norms in learning and memory, with impairment rates exceeding 20% in these domains. These results emphasize the limitations of existing norms and the critical role of local controls in validating their applicability to contemporary populations.

The observed differences between existing norms and local controls may reflect two main factors. First, longitudinal differences: norms established 10 to 20 years ago may not account for significant lifestyle changes, such as increased sedentary behavior, reliance on electronic devices, and reduced physical exercise. These lifestyle changes have been linked to declines in memory and attention, contributing to the observed discrepancies (24). At the same time, the relatively high attention/working memory performance of the PLWoH group may reflect rising educational attainment and stronger test-taking familiarity in recent urban cohorts, particularly in cognitively demanding tasks like the PASAT. Second, regional differences: norms derived from multiple cities (e.g., Shanghai, Kunming, Hong Kong, and Beijing) encompass linguistic, cultural, and socioeconomic variations, as well as differences in HIV-related factors such as transmission routes and antiretroviral regimens. All these factors may have contributed to baseline disparities. Environmental factors like air quality and temperature could also influence overall cognitive performance (25). These findings highlight the clinical necessity of updating and localizing cognitive test norms.

The contrast between the two scoring approaches was also reflected in the Frascati-defined classifications. Using historical norms, nearly half of PLWH (46%) and over a third of PLWoH (36%) met the criteria for ANI. In contrast, when using local sample-based T-scores, the corresponding rates dropped to 6 and 1%, respectively. This sharp discrepancy supports earlier concerns that ANI prevalence may be inflated when outdated or mismatched norms are applied (26). For example, a recent large-scale study using internal cohort norms estimated ANI prevalence at 17%, underscoring how diagnostic outcomes depend heavily on the chosen reference group (27). While these findings reinforce the importance of localized standards, they should be interpreted cautiously, as our local control group was not designed to serve as a normative reference.

Compared to other studies using local controls, this study found fewer cognitive deficits among PLWH, with impairments largely confined to attention/working memory and executive function. For instance, Heaton et al. (18) analyzed data from rural Anhui and reported deficits across seven cognitive domains among PLWH compared to local controls, and Qin et al. (28) observed similar findings in urban Hunan. The narrower range of deficits in this study may reflect advancements in HIV diagnosis and antiretroviral therapy over the past two decades, including earlier detection and improved cognitive outcomes due to timely intervention. Additionally, the relatively younger age of participants in this study (mean age: 36 years) compared to previous studies (41, 61, and 46 years, respectively) may also account for the differences, as cognitive impairment rates among PLWH are known to increase with age (6).

This study has several limitations. First, the sample-based T-scores used for normalization were derived from all participants rather than confined to PLWoH, which, while maximizing sample size, is not a rigorous approach. However, our main conclusions were based on group comparisons, which were not fundamentally affected by this process. Second, the average age of PLWoH was lower than that of PLWH. Although regression analyses controlled for age and related factors, as done in previous studies (29), residual age-related effects may still contribute to differences in attention/working memory. Third, the relatively small sample size of the PLWoH group precludes the establishment of robust new norms. Future studies should aim to collect more extensive data, targeting a minimum of 100 participants per age group within a short time frame, to establish robust and regionally relevant norms (30). Finally, clinical records were only available for a subset of PLWH due to accessibility limitations, restricting the inclusion of clinical variables in group-level analyses. This may limit the interpretation of cognitive performance in relation to disease severity.

This study demonstrates that PLWH living in Shanghai primarily exhibit cognitive impairments in attention/working memory and executive function. Although impairments in learning, memory, and motor domains were observed when compared to preexisting norms from other cities, these deficits may reflect broader declines in these cognitive domains within the general population, potentially driven by non-HIV-related factors. Such declines may result from environmental pollution, lifestyle changes, and other non-HIV-related factors. To avoid misdiagnoses and ineffective interventions caused by outdated or inappropriate norms, future research should use well-matched local HIV-negative controls or develop updated and localized cognitive test norms.

## Data Availability

The data analyzed in this study is subject to the following licenses/restrictions: the dataset used in this research was collected as part of other ongoing projects and contains sensitive information that requires careful management. Requests to access these datasets should be directed to danchao.cai@outlook.com.
